# Metabolic and molecular responses of human patellar tendon to concentric- and eccentric-type exercise in youth and older age

**DOI:** 10.1007/s11357-022-00636-x

**Published:** 2022-08-11

**Authors:** Hannah Crossland, Matthew S. Brook, Jonathan I. Quinlan, Martino V. Franchi, Bethan E. Phillips, Daniel J. Wilkinson, Constantinos N. Maganaris, Paul L. Greenhaff, Nathaniel J. Szewczyk, Kenneth Smith, Marco V. Narici, Philip J. Atherton

**Affiliations:** 1 MRC Versus Arthritis Centre for Musculoskeletal Ageing Research and NIHR Nottingham Biomedical Research Centre, University of Nottingham, Royal Derby Hospital Centre, Derby, DE22 3DT UK; 2 School of Sport, Exercise and Rehabilitation Sciences, University of Birmingham, Birmingham, UK; 3 3National Institute for Health Research, Birmingham Biomedical Research Centre at University Hospitals Birmingham NHS Foundation Trust, Birmingham, UK; 4 Department of Biomedical Sciences, University of Padova, Padua, Italy; 5 School of Sport and Exercise Sciences, Liverpool John Moores University, Liverpool, UK; 6 Ohio Musculoskeletal and Neurological Institute (OMNI) and Department of Biomedical Sciences, Ohio University, Athens, OH 45701 USA; 7 CIR-MYO Myology Center, University of Padova, Padua, Italy

**Keywords:** Tendon, Protein synthesis, Collagen, D2O, Training

## Abstract

Exercise training can induce adaptive changes to tendon tissue both structurally and mechanically; however, the underlying compositional changes that contribute to these alterations remain uncertain in humans, particularly in the context of the ageing tendon. The aims of the present study were to determine the molecular changes with ageing in patellar tendons in humans, as well as the responses to exercise and exercise type (eccentric (ECC) and concentric (CON)) in young and old patellar tendon. Healthy younger males (age 23.5 ± 6.1 years; *n* = 27) and older males (age 68.5 ± 1.9 years; *n* = 27) undertook 8 weeks of CON or ECC training (3 times per week; at 60% of 1 repetition maximum (1RM)) or no training. Subjects consumed D_2_O throughout the protocol and tendon biopsies were collected after 4 and 8 weeks for measurement of fractional synthetic rates (FSR) of tendon protein synthesis and gene expression. There were increases in tendon protein synthesis following 4 weeks of CON and ECC training (*P* < 0.01; main effect by ANOVA), with no differences observed between young and old males, or training type. At the transcriptional level however, ECC in young adults generally induced greater responses of collagen and extracellular matrix-related genes than CON, while older individuals had reduced gene expression responses to training. Different training types did not appear to induce differential tendon responses in terms of protein synthesis, and while tendons from older adults exhibited different transcriptional responses to younger individuals, protein turnover changes with training were similar for both age groups.

**Supplementary information:**

The online version contains supplementary material available at 10.1007/s11357-022-00636-x.

## Introduction

Ageing is associated with progressive structural and mechanical changes to collagen-rich tissues such as tendon [1], and in elderly individuals, this can have negative impacts on tissue function and contribute to functional disabilities and injuries [2–4]. Tendon tissues are predominantly composed of collagen and non-collagenous extracellular matrix (ECM) components including proteoglycans, the turnover of which is regulated by matrix metalloproteinases (MMPs) and tissue inhibitors of metalloproteinases (TIMPs). Resistance exercise (RE) appears to attenuate age-related functional declines in tendons by altering tendon mechanical properties [2]; however, the molecular changes that underlie these responses are uncertain.

The majority of data regarding the effects of ageing on tendon structural and functional properties have been generated from in vitro and animal models. Tenocytes cultured from mice of different ages revealed a decrease in rates of proliferation with age and a reduction in expression of stem cell markers [5, 6]. In rat tendons, Kostrominova et al. [7] observed decreased gene expression of collagen types I, III and V, as well as key proteoglycans and ECM-related proteins, with increasing age. At the structural level, ageing patellar tendons from mice were demonstrated to have deterioration of viscoelastic properties, with reduced collagen fibre alignment and altered tenocyte shape [8]. In humans, transcriptomic analysis of Achilles tendons with ageing found distinct differences between old and young tendons, with altered expression of genes important in cell growth and cell cycle pathways [9].

While it is known that RE can result in tendon tissue adaptive changes both structurally and mechanically [10], the underlying regulatory factors that contribute to these changes remain uncertain, particularly in the context of the ageing tendon. In humans, acute (4 h) RE resulted in reduced expression of collagen type I and III and MMPs [11], while additional studies in human tendon have shown that acute exercise induced few changes in gene expression (related to collagen, growth factors and matrix proteins) compared to the changes in muscle [12]. In relation to ageing, a recent study in older rats demonstrated that resistance training increased growth factor expression and proteoglycan content in tendons of old rats [13], while 10 weeks of uphill running resulted in reduced tendon stiffness and increased gene expression of collagen turnover related genes in Achilles tendons of older mice [14].

Studies have also focused on the effects that different exercise intensities and types may have on tendon adaptation. In the work of Xu et al. [15], moderate intensity treadmill running induced collagen synthesis and collagen fibril organisation, while high intensity running appeared to cause collagen degradation and disturbance in rat Achilles tendons. Two further studies compared 4 days of concentric (CON) and eccentric (ECC) training in female rats (as the greater absolute mechanical load in ECC vs. CON could produce differential anatomical, cellular and molecular responses), observing that acute exercise upregulated IGF-1 isoforms in Achilles tendon, as well as genes related to collagen synthesis and processing; however, no difference between contraction mode was observed [16, 17]. In humans, studies have suggested that different contraction types and intensities can differentially influence tendon mechanical properties in young and older adults [18, 19]; however, the cellular/molecular factors underlying those differences require further study.

The aims of the present study were to determine the molecular changes with ageing in tendons in humans (specifically, at the level of tendon protein synthesis and gene expression changes), as well as the subsequent impacts of 4 and 8 weeks of training. ECC and CON training types were compared in order to evaluate whether there were any differences in tendon remodelling based on training mode. Our hypothesis was that older tendons would exhibit differences in molecular properties (including collagen and ECM-related molecular changes), and that training would induce tendon cellular adaptations in both age groups, but that these changes would be attenuated in older individuals.

## Methods

### Ethical approval, participants, study protocol and sample collection

Fifty-four healthy, recreationally active individuals were recruited, 27 of which were younger males (age (mean ± SD): 23.5 ± 6.1 years; body weight: 74.4 ± 13.2 kg) and 27 older males (age: 68.5 ± 1.9 years; body weight: 78.7 ± 9.4 kg). All participants underwent a full medical screening prior to enrolment, whereby those with any musculoskeletal, metabolic, respiratory, neurological or cardiovascular medical conditions were excluded from the study. None of the participants utilised in this study was deemed as frail or having had a history of falls. All participants provided written informed consent to this study. The study was approved by the University of Nottingham Ethics Committee and was performed in accordance with the Declaration of Helsinki (approval number B13032014 SoMSGEM).

For both younger and older males, participants were randomised into 3 groups: eccentric (ECC; *n* = 9), concentric (CON; *n* = 9) or control (*n* = 9). As tendon biopsy limitations meant pre-training samples could not be collected from each individual, inclusion of the control (non-trained) group provided baseline measures and basal enrichment measurements. CON and ECC groups underwent 8 weeks training with 3 sessions per week, utilising a custom leg press machine, which enabled the isolation of CON and ECC only contractions (a set-up that has previously been described in detail [20]). The first 4 weeks was bilateral and was switched to unilateral for final 4 weeks, due to the tendon biopsy acquired. Each exercise bout consisted of 4 sets of 12–15 repetitions at 60% load of either CON or ECC 1 repetition maximum (1RM), with 2-min rest between sets. Training load was monitored and progressively increased according to changes in ECC or CON 1RM. Functional outcomes of the training responses, and tendon biomechanical properties for this study, have previously been described in Quinlan et al. [21]. Participants were provided with D_2_O which was taken as a bolus of < 250 ml (based on body weight) followed by < 100 ml per week. Two saliva samples were collected each week for determination of body water enrichment. At the 4- and 8-week timepoints, patellar tendon biopsies were collected from the participants (60 min after a training session). Briefly, under sterile conditions and ultrasound guided, an automatic disposable 14 G Bard Monopty Biopsy Instrument (Bard Inc., Covington, GA, USA) was utilised to obtain ~ 10 mg of tissue from the proximal patellar tendon via a single pass (Supporting Fig. 1). The tissue was consequently snap frozen in an RNase-free tube and placed in a − 80 °C freezer for future analysis.

**Fig. 1:**
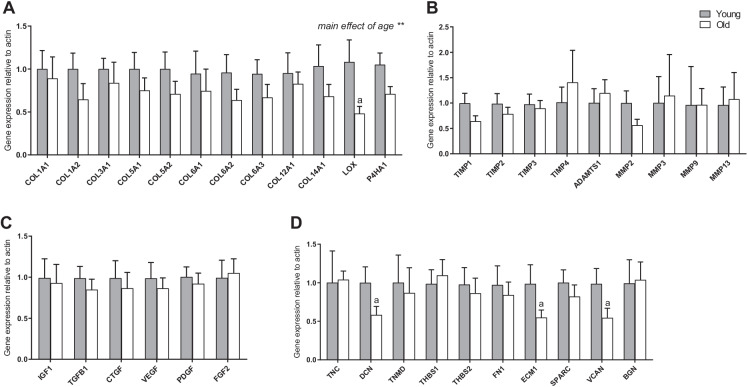
Expression of extracellular matrix (ECM)-related and regulatory growth factor genes between young and old individuals in tendon tissue. Expression of genes related to collagen proteins (**A**), ECM remodelling (**B**), regulatory growth factors (**C**), and structural ECM-related proteins and proteoglycans (**D**) were compared between old and young untrained individuals in tendon tissue, with data normalised to actin expression and presented as fold change versus young group (*n* = 9 per group). Results are displayed as mean + SEM. ^a^*P* < 0.05 versus young group

### RNA extraction, cDNA synthesis and real-time PCR

Portions of tendon samples (weights ~ 4–8 mg) were placed in 2-ml tubes along with 500 µl ice-cold TRI Reagent® (Sigma-Aldrich, UK) and 5-mm stainless steel beads (Qiagen, UK). Tissue was lysed by homogenising for 2–3 rounds of 30 s using a TissueLyser II (Qiagen, UK), until tissue was visibly homogenised. Samples were kept on ice in-between each round of homogenisation.

Following homogenisation, RNA extraction was carried out according to the TRI Reagent® manufacturer’s protocol (Sigma-Aldrich, UK). The RNA pellet was resuspended in 10 µl RNase-free water, and RNA quantity and quality (260:280 nm and 260:230 nm ratios) were determined using a NanoDrop™ 2000 (Thermo Fisher Scientific). At the stage of phase separation, the lower (protein-containing) phase was kept for protein isolation to be used for the stable isotope enrichment analyses (see below).

RNA (500 ng) was reverse transcribed using the high-capacity cDNA synthesis kit (Applied Biosystems, Thermo Fisher Scientific), which was diluted 1:5 using RNase-free water. For targeted real-time PCR measurements, 1 µl of cDNA was loaded into 384-well plates (in duplicate) with SYBR™ Select Master Mix (Applied Biosystems, Thermo Fisher Scientific) and gene-specific primers (for list of primers used, see Table 1). Genes were selected to represent a range of biological pathways related to structural and regulatory proteins in tendon. The ΔΔCt method [22] was used to quantify mRNA expression, with *ACTB* expression being used for normalisation (during initial tests, a total of 5 potential housekeeping genes were assessed, including *PPIA*, *ACTB*, *RPL13A*, *TBP* and *UBC*, and *ACTB* was the most stable gene across age and training type).

**Table 1:** Sequences for primers used in the study

Gene symbol	Gene name	Forward (5′ to 3′)	Reverse (5′ to 3′)
*COL1A1*	Collagen Type I Alpha 1 Chain	ACTGGTGAGACCTGCGTGTA	GCCGCCATACTCGAACTGGA
*COL1A2*	Collagen Type I Alpha 2 Chain	CAGCCGGAGATAGAGGACCA	CAGCAAAGTTCCCACCGAGA
*COL3A1*	Collagen Type III Alpha 1 Chain	TGGAGGATGGTTGCACGAAA	ACAGCCTTGCGTGTTCGATA
*COL5A1*	Collagen Type V Alpha 1 Chain	CTGTGCTACCAAGAAAGGCTA	CTAGCCCATGAAGCAAGC
*COL5A2*	Collagen Type V Alpha 2 Chain	ACATGATGGCAAACTGGGCG	TTCACCATATCCTTCATCCTCGT
*COL6A1*	Collagen Type VI Alpha 1 Chain	TAAAGGCTACCGAGGCGATG	GCCGTCTTCTCCCCTTTCAC
*COL6A2*	Collagen Type VI Alpha 2 Chain	CCTCGGGACCAGGACTTCAG	GGTAGTGTCCGGCGAGATG
*COL6A3*	Collagen Type VI Alpha 3 Chain	CAGGTTTGCTCAGGGGTTCA	CAGCCGCSCCSTTTTTGACA
*COL12A1*	Collagen Type XII Alpha 1 Chain	CGGGGGATGACAGAAGACTG	ACCAGCCCTAAGTTCCTCCA
*COL14A1*	Collagen Type XIV Alpha 1 Chain	CCTGTCAGTGTTCCTGGTCC	CCATTCCGCTGAGGAGGTTT
*LOX*	Lysyl Oxidase	CTGAAGGCCACAAAGCAAGTT	AGGACTCAATCCCTGTGTGT
*P4HA1*	Prolyl 4-Hydroxylase Subunit Alpha 1	ACCTAGCAAAACCAAGGCTGA	TTCATAGCCAGAGAGCCAGG
*TIMP1*	TIMP Metallopeptidase Inhibitor 1	CATCCGGTTCGTCTACACCC	TGCAGTTTTCCAGCAATGAGA
*TIMP2*	TIMP Metallopeptidase Inhibitor 2	GTTTATCTACACGGCCCCCT	TCGGCCTTTCCTGCAATGAG
*TIMP3*	TIMP Metallopeptidase Inhibitor 3	ACCGAGGCTTCACCAAGATG	CCATCATAGACGCGACCTGT
*TIMP4*	TIMP Metallopeptidase Inhibitor 4	CTGCCAAATCACCACCTGCTAC	GGTGCCGTCAACATGCTTC
*ADAMTS1*	ADAM Metallopeptidase With Thrombospondin Type 1 Motif 1	TGCAGCCCAAGGTTGTAGAT	AGATCCATTTCCCCCGCAAA
*MMP2*	Matrix Metallopeptidase 2	ACAAAGAGTTGGCAGTGCAATA	TCTGGGGCAGTCCAAAGAAC
*MMP3*	Matrix Metallopeptidase 3	CACTCACAGACCTGACTCGG	AGGGGGAGGTCCATAGAGGG
*MMP9*	Matrix Metallopeptidase 9	TTCTGCCCGGACCAAGGATA	ACATAGGGTACATGAGCGCC
*MMP13*	Matrix Metallopeptidase 13	GACCCTGGAGCACTCATGTT	CCTGGACCATAGAGAGACTGG
*IGF1*	Insulin Like Growth Factor 1	AAATCAGCAGTCTTCCAACCC	GTGTGCATCTTCACCTTCAAGAAA
*TGFB1*	Transforming Growth Factor Beta 1	AGTGGACATCAACGGGTTCAC	GAAGCAGGAAAGGCCGGTT
*CTGF*	Connective Tissue Growth Factor	ACCAATGACAACGCCTCCTG	TGCCCTTCTTAATGTTCTCTTCC
*VEGFA*	Vascular Endothelial Growth Factor A	ACAACAAATGTGAATGCAGACCA	TACCGGGATTTCTTGCGCTT
*PDGFA*	Platelet Derived Growth Factor Subunit A	CCGTAGGGAGTGAGGATTCTT	ACAGCTTCCTCGATGCTTCT
*FGF2*	Fibroblast Growth Factor 2	GCTGTACTGCAAAAACGGGG	TAGCTTGATGTGAGGGTCGC
*TNC*	Tenascin C	GCCTCCACAGGGGAAACTC	GTACTCATAGGCCCCTTCTGG
*DCN*	Decorin	CTAGTCACAGAGCAGCACCT	CAGGGAACCTTTTAATCCGGGAA
*TNMD*	Tenomodulin	GTGTTTGGTATCCTGGCCCT	GTGCTCCATGTCATAGGCTTTT
*THBS1*	Thrombospondin 1	GCATCCGCAAAGTGACTGAA	AGTGCAGCTATCAACAGTCCA
*THBS2*	Thrombospondin 2	CCAGGGAGCTGAGATCAACG	CACGTACTCGGTGGTGACAT
*FN1*	Fibronectin 1	CTGACAGCTCATCCGTGGTT	TTGGTGGGCTGACATTCTCC
*ECM1*	Extracellular Matrix Protein 1	GGGACAGAGTCAAGTGCAGC	TTGGGCAGGTAGCAGCTTTT
*SPARC*	Secreted Protein Acidic And Cysteine Rich	GAACCACCACTGCAAACACG	TGTCATTGCTGCACACCTTC
*VCAN*	Versican	AGGTGGTCTACTTGGGGTGA	TGGTTGTAGCCTCTTTAGGTTT
*BGN*	Biglycan	GACACACCGGACAGATAGACG	CATGGCGGATGGACCTGGAG

### Protein extraction, hydrolysis and determination of protein-bound alanine enrichment

Due to the limited size of tendon biopsies, we aimed to measure protein-bound alanine enrichment from the protein fraction that was generated during the TRI Reagent® RNA extractions. Initial tests determined that comparable fractional synthetic rates (FSR) were generated using this method and protein extraction methods that have previously been published ([23]; data not shown). In addition, initial tests aimed to fractionate the samples into alkali-soluble and alkali-insoluble parts; however, tests with human tendons did not yield consistent results/sufficient alkali soluble protein for mass spectrometric analysis. Protein was extracted from the lower phase of TRI Reagent® extractions by adding isopropanol and centrifuging for 10 min at 12,000 × *g*. Protein-containing pellets were washed twice with ethanol, and then protein was hydrolysed by incubating in 0.1 mol L^−1^ HCl in Dowex H^+^ resin overnight at 110 °C. Samples were isolated by passing through Dowex resin columns, eluted with 2 mol L^−1^ NH4OH and dried down. Amino acids were derivatised as their N-methoxycarbonyl methyl esters. Protein-bound alanine enrichment was determined by gas chromatography-pyrolysis-isotope ratio mass spectrometry (Delta V Advantage; Thermo Scientific). Within the same sample run, the samples were used to determine the ratio of hydroxyproline/proline.

Protein synthesis was calculated from the incorporation of deuterium-labelled alanine into protein, with enrichment of body water as precursor labelling, as previously described [24]. Fractional synthetic rate (FSR) was calculated using the following equation:

$$\mathrm{FSR}\left(\%/\mathrm{day}\right)=-In\left[\frac{1-\left[\frac{{(APE}_{Ala})}{({APE}_P)}\right]}t\right],$$

where *t* is time in days, APE_Ala_ is the deuterium enrichment of protein-bound alanine and APE_P_ is the average enrichment of the body water precursor, i.e. saliva, over the time between biopsies. Since there were no biopsies collected at time = 0, additional tendon biopsies were collected from individuals who did not receive D_2_O, and these were used for baseline enrichment.

### Body water enrichment

Enrichment of body water was determined using 50 µl saliva which was evaporated onto inverted autosampler vials (4 h at 100 °C) and then placed upright onto ice. Enrichment of body water was determined by injecting into a high-temperature conversion elemental analyser (Thermo Finnigan, Thermo Scientific, Hemel Hempstead, UK) connected to a Delta V Advantage Isotope Ratio Mass Spectrometer (Thermo Finnigan, Thermo Scientific).

### Tendon cross sectional area (CSA)

Transversal magnetic resonance imaging (MRI) scans were performed at each timepoint for all training groups via a 3 T scanner. Participants were lying supine, with the knee joint fixed at 180°. Slice thickness was set at 3 mm with no interslice gap. Tendon CSA values were calculated to correspond to the biopsy site (Supporting Fig. 1). A total of three CSA images were manually measured via offline digital analysis software (OsiriX Lite 9.5.2, Pixmeo SARL), and an averaged value was generated for further analysis. Note that one elderly ECC was unable to have an MRI scan due to medical reasons and hence *n* = 8 for the old ECC group, whereas *n* = 9 for all others.

### Statistical analyses

For gene expression analyses, genes were sub-categorised based on ontology, and 1-way ANOVA was used to assess main effects of each training type for each age group and timepoint. Sidak’s multiple comparison tests were used to determine any individual changes in gene expression between groups (*P* < 0.05 was considered statistically significant). For protein synthesis analyses, exercise and age comparisons were made using two-way ANOVA with Sidak’s multiple comparisons tests. Statistical tests were performed using Prism version 7 (GraphPad Software Inc., San Diego, CA, USA).

## Results

### Gene expression comparisons in tendon tissue between young and older males

An age comparison of genes related to tendon structure, remodelling and turnover was evaluated in untrained young and old individuals. Individually, there were no significant differences between young and older males for collagen encoding genes; however, there was an overall (main effect by ANOVA) downregulation of collagen genes with age (*P* < 0.05 vs. young), and there was a significant decrease in *LOX* expression in older males (0.5 ± 0.1-fold; *P* < 0.05 vs. young; Fig. 1A). There were no differences between young and older males for genes involved in ECM remodelling or regulatory growth factors (Fig. 1B, C, respectively). In terms of tendon structural-related genes, there were specific decreases in *DCN* (0.6 ± 0.1-fold; *P* < 0.05 vs. young), *ECM1* (0.5 ± 0.1-fold; *P* < 0.05 vs. young) and *VCAN* (0.5 ± 0.1-fold; *P* < 0.05 vs. young) mRNA expression in older individuals (Fig. 1D).

### Protein synthesis and collagen proline hydroxylation in tendon tissue following 4- and 8-week eccentric or concentric training in young and older males

**Fig. 2:**
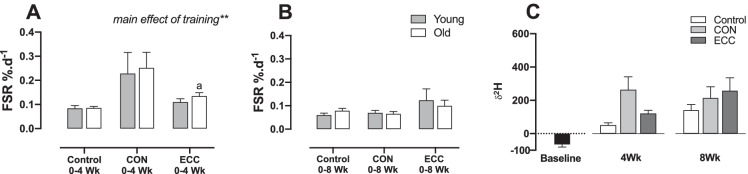
Fractional synthetic rates (FSR) of protein synthesis in young and old individuals following 4- and 8-week eccentric or concentric training in tendon tissue. Tendon protein synthesis rates with 4- (**A**) and 8- (**B**) week concentric training or eccentric training in young and older males, compared to control group. *n* = 9 per group. ^a^*P* < 0.05 versus control group. Tendon protein ^2^H incorporation at 4 and 8 weeks in young and old combined (**C**). Baseline (black bar) represents ^2^H levels in tendon prior to D2O consumption

There were no differences in tendon protein synthesis between young and older males in the control group (Fig. 2A). Following 4 weeks of CON training, there was a main effect (*P* < 0.01 by ANOVA) of training (increase) on tendon protein synthesis rates for both the young and old groups (0.23 ± 0.09%/day vs. 0.084 ± 0.012%/day in young CON vs. young controls; 0.25 ± 0.06%/day vs. 0.085 ± 0.007%/day in old CON vs. old controls; Fig. 2A), whereas by 8 weeks of CON training, protein synthesis rates of tendon were no different from the control group, for both young and old adults (Fig. 2B). Following 4 weeks of ECC training, there was a main effect (*P* < 0.01 by ANOVA) of exercise (increase) on tendon protein synthesis rates for both the young and old groups (Fig. 2A), and post hoc testing showed a significant increase in FSR in the old group (0.13 ± 0.014%/day vs. 0.085 ± 0.007%/day; *P* < 0.05). By 8 weeks of ECC training, protein synthesis rates of tendon were no different from the control group, for both young and old adults (Fig. 2B).

**Fig. 3:**
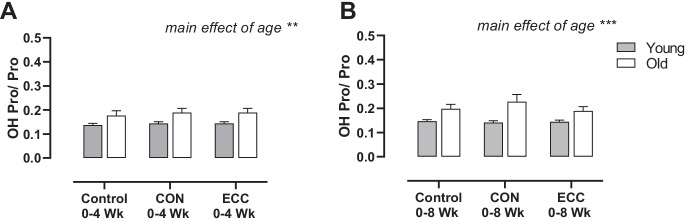
Hydroxyproline to proline ratios in young and old individuals following 4- and 8-week eccentric or concentric training in tendon tissue. Ratio of hydroxyproline to proline in tendon tissue with 4- (**A**) and 8- (**B**) week concentric training or eccentric training in young and older males, compared to control group. *n* = 9 per group

The ratio of hydroxyproline to proline in tendon tissue was assessed with training and across age groups. There were no significant effects of either training type at 4 or 8 weeks (Fig. 3); however, there was a main effect of an increase in hydroxylated proline with increased age (*P* < 0.01 by ANOVA; Fig. 3).

### Gene expression in tendon tissue following 4- and 8-week eccentric or concentric training in young males

**Fig. 4:**
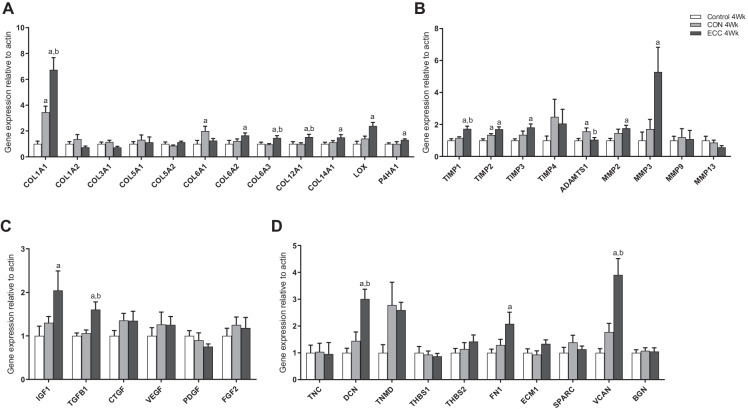
Expression of extracellular matrix (ECM)-related and regulatory growth factor genes in young individuals following 4-week eccentric (ECC) or concentric (CON) training in tendon tissue. Expression of genes related to collagen proteins (**A**), ECM remodelling (**B**), regulatory growth factors (**C**) and structural ECM-related proteins and proteoglycans (D) were compared in young individuals following 4-week ECC or CON training in tendon tissue, with data normalised to actin expression and presented as fold change versus controls (*n* = 9 per group). Results are displayed as mean + SEM. ^a^*P* < 0.05 versus control group. ^b^*P* < 0.05 versus CON group

Genes encoding collagen proteins and factors that regulate collagen cross-linking were assessed in tendons of young males following 4 weeks ECC or CON training. Following 4 weeks CON training, there were increases in *COL1A1* (3.4 ± 0.5-fold; *P* < 0.001 vs. control) and *COL6A1* (2.0 ± 0.4-fold; *P* < 0.05 vs. control) expression relative to young controls, while other collagen genes measured were unchanged (Fig. 4A). With ECC training, however, increases in mRNA expression of *COL1A1*, *COL6A2*, *COL6A3*, *COL12A1*, *COL14A1*, *LOX* and *P4HA1* were observed, relative to untrained individuals (Fig. 4A). *COL1A1*, *COL6A2*, *COL6A3*, *COL12A1* and *LOX* were also all upregulated with ECC relative to the CON group. Following 8 weeks of training, there were no changes in gene expression in the CON group, and in contrast to the changes observed after 4 weeks of training, the majority of genes were unchanged with ECC training, with only an increase in *COL1A1* expression observed (2.4 ± 0.4-fold; *P* < 0.05 vs. control; Supporting Fig. 2).

Gene expression of selected TIMPs and MMPs (encoding key ECM remodelling proteins) were also measured with training in patellar tendon tissue of young individuals. Expression of *TIMP2* mRNA was increased with CON training (1.3 ± 0.1-fold; *P* < 0.05 vs. control), while *TIMP1*, *TIMP3* and *TIMP4*, and all MMPs measured, were unaltered (Fig. 4B). With ECC however, *TIMP1*, *TIMP2* and *TIMP4* mRNA were all elevated compared to control, while increases in *MMP2* and *MMP3* gene expression were also observed (Fig. 4B). Expression of *ADAMTS1*, however, was increased with CON training (1.6 ± 0.2-fold; *P* < 0.05 vs. control), with no change observed in the ECC group.

Regulatory growth factor genes were also assessed in tendons of young individuals following 4-week CON or ECC training. None of the measured growth factor genes was altered with CON training; however, *IGF1* and *TGFB1* were significantly increased with ECC training (2.0 ± 0.5-fold; *P* < 0.05 vs. control and 1.6 ± 0.2-fold; *P* < 0.01 vs. control, respectively; Fig. 4C). There were, however, no changes in *CTGF*, *VEGF*, *PDGF* or *FGF2* in tendons of trained individuals relative to controls (Fig. 4C).

Genes encoding structural ECM-related proteins and proteoglycans were measured, with no observed changes seen with 4-week CON training. With ECC however, increases in *DCN* (3.0 ± 0.4-fold; *P* < 0.001 vs. Control), *FN1* (2.1 ± 0.4-fold; *P* < 0.05 vs. Control) and *VCAN* (3.9 ± 0.6-fold; *P* < 0.001 vs. Control) were observed in tendon relative to untrained controls (Fig. 4D).

### Gene expression in tendon tissue following 4- and 8-week eccentric or concentric training in older males

**Fig. 5:**
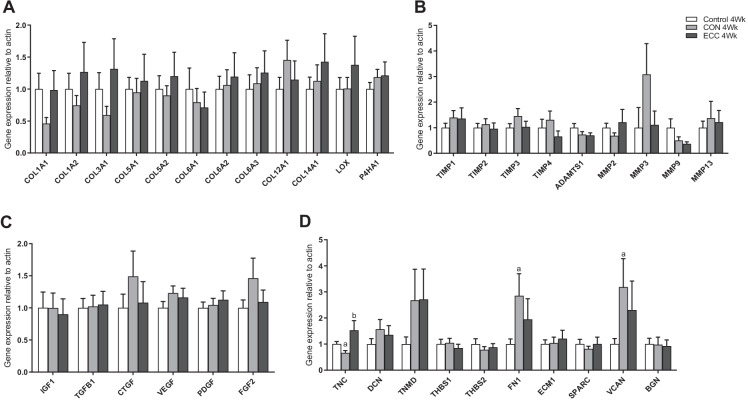
Expression of extracellular matrix (ECM)-related and regulatory growth factor genes in older individuals following 4-week eccentric (ECC) or concentric (CON) training in tendon tissue. Expression of genes related to collagen proteins (**A**), ECM remodelling (**B**), regulatory growth factors (**C**) and structural ECM-related proteins and proteoglycans (**D**) were compared in older individuals following 4-week ECC or CON training in tendon tissue, with data normalized to actin expression and presented as fold change versus controls (*n* = 9 per group). Results are displayed as mean + SEM. ^a^*P* < 0.05 versus control group. ^b^*P* < 0.05 versus CON group

Gene expression changes were assessed in tendons of older males following 4 weeks ECC or CON training. There were no changes in genes encoding collagen proteins for either training type at 4 weeks (Fig. 5A). Similarly, genes encoding ECM remodelling proteins and regulatory growth factors were unaltered with either training type in older males after 4 weeks (Fig. 5B, C).

In terms of genes encoding structural proteins and proteoglycans, 4 weeks of CON training resulted in an increase in *FN1* (2.8 ± 0.8-fold; *P* < 0.05 vs. control) and *VCAN* (3.1 ± 1.1-fold; *P* < 0.05 vs. control) expression, while *TNC* expression was decreased (0.6 ± 0.1-fold; *P* < 0.05 vs. control), and other measured genes remained unchanged (Fig. 5D). With ECC training, no genes encoding structural-related ECM proteins were altered after 4 weeks.

At 8 weeks of training, there were no changes in gene expression for most targets analysed, although there was a decrease in *THBS2* and *ECM1* mRNA with CON training and a significant decrease in *THBS1* expression (0.5 ± 0.1-fold; *P* < 0.05 vs. control) following 8-week ECC training (Supporting Fig. 3).

### Proximal CSA changes in response to 4 and 8 weeks of eccentric or concentric training

**Fig. 6:**
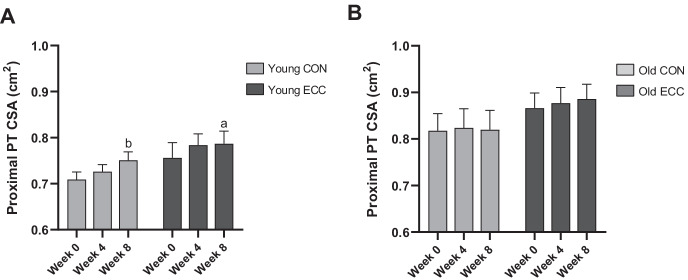
Proximal tendon cross-sectional area (CSA) in both young and older individuals following 4- and 8-week eccentric (ECC) or concentric (CON) training. The tendon proximal CSA in young (**A**) and old (**B**) individuals following ECC or CON training (*n* = 9 for all except Old ECC, *n* = 8). a = *P* < 0.05 vs. baseline, b = *P* < 0.01 vs. baseline

Proximal tendon CSA was significantly increased following 8 weeks of CON (0.71 ± 0.04cm^2^ vs. 0.75 ± 0.05cm^2^, *P* < 0.01) and ECC training in the young groups (0.75 ± 0.09cm^2^ vs. 0.78 ± 0.08cm^2^, *P* < 0.05) (Fig. 6). However, no changes were observed in either CON or ECC elderly groups.

## Discussion

Although it is known that exercise training can result in adaptive changes to tendon tissue at the structural, functional [25] and metabolic level [26], the underlying regulatory factors that contribute to these alterations are poorly understood in humans, particularly in the ageing tendon. In the present study, we aimed to examine the molecular changes with ageing in patellar tendons in humans, as well as the impact of 4- and 8-week eccentric (ECC) and concentric (CON) training. We found that while ECC appeared to induce a greater remodelling response of tendon than CON at the level of gene expression, this did not impact on tendon protein turnover, since increases in protein synthesis were comparable between training types in young and older individuals. Furthermore, despite transcriptional changes indicating reduced responsiveness of training in older adults, protein synthesis responses with training were similar for both age groups. These findings provide novel insights into tendon adaptation to mechanical loading at the molecular level with human ageing.

### Age differences in tendon protein metabolism and gene expression

In the present study, we sought to determine basal age-related differences in tendon properties in terms of protein metabolism and gene expression. In the group of non-exercised individuals, there was no difference in protein turnover throughout the period studied. In terms of mRNA changes, there was an overall downregulation of tendon collagen genes, and specific proteoglycan genes (*DCN*, *ECM1* and *VCAN*) in older age, which is in general agreement to previous work in rat tendons with ageing [7]. While these changes did not appear to impact on global protein turnover, they could feasibly contribute to specific structural and/or mechanical changes in tendons of older individuals.

One observed age-related difference was the increase in proline hydroxylation observed in tendons of older males (but no alterations with training), an observation that has previously been reported [23]. Proline hydroxylation is an important component of collagen formation since it provides structural stability of the triple helix [27, 28]. Proline hydroxylation may also have additional biological functions, since many collagen-interacting proteins contain hydroxyproline functional motifs [28]. Thus, the potential impacts of increased proline hydroxylation in tendon collagen, and how this may have mechanical/functional consequences, require further study. We recently observed, in these same individuals [20], that while there were adaptations in the tendon with training in both the older and younger groups, in terms of changes in tendon biomechanical properties (tendon stiffness and Young’s modulus), the older tendon appeared to take longer to maximally respond. While the underlying factors that contribute to this observation are not clear, it is possible that the age-related differences in collagen-related gene expression and hydroxyproline could (at least partially) influence tendon mechanical properties.

### Tendon tissue remodelling following 4- and 8-week eccentric or concentric training in young males

Tendon tissue changes in protein synthesis and gene expression were measured in young males undergoing 4 and 8 weeks of ECC or CON exercise, relative to untrained controls. After 4 weeks of training, tendon protein synthesis was increased in young males, with no significant differences between training types. Tendon collagen synthesis has been previously demonstrated to increase in response to acute exercise in healthy young males [29], and longer-term training [30]; however, the effects of specific contraction types on tendon protein synthesis remain poorly understood. In skeletal muscle, 4-week ECC or CON training did not result in any differences in protein synthesis [31] and not even after 8 weeks of the same training program [32], while a study in rats reported that tendons were not responsive to contraction type in terms of transcriptional changes in collagen and anabolic growth factor genes [16, 17]. Our study provides further insight into the adaptive responses of tendon tissue with training, specifically, that in terms of protein synthesis, tendon may be unresponsive to contraction type in humans, but shown to be responsive to contraction load.

Gene expression changes (related to collagen, growth factor, proteoglycan and ECM remodelling genes) were also evaluated in tendon tissue with ECC and CON training in young individuals, with the aim of further understanding the molecular changes underlying tendon remodelling with exercise. We found that changes with exercise appeared to occur early in tendon (with few changes seen after 8 weeks of training), while ECC appeared to induce greater remodelling responses in tendon than CON (in terms of gene expression). A previous study in humans demonstrated that acute RE induced changes in tendon gene expression of collagen and matrix remodelling genes [11]. In that study, there were no observed changes in proteoglycan genes, although previous work has demonstrated tendon proteoglycans to be responsive to mechanical loading [33, 34]. Several MMP/TIMP genes were also induced after 4 weeks of ECC training in younger adults, including MMP2 and MMP3. MMP proteins are responsible for ECM breakdown, and MMP2 and MMP3 have been previously shown to be upregulated with mechanical stimuli [35, 36]. Whether the different contraction type with ECC exercise induced greater ECM breakdown in the tendon requires further evaluation. Overall, ECC appeared to induce a greater gene expression response than CON training in young individuals, but these differences were not reflected in overall rates of protein synthesis. It should be noted that the gene expression changes reflect a single point in time, while the protein synthesis measures represent cumulative changes over a 4-/8-week period. However, while ECC appeared to have a greater impact on gene expression, changes in tendon CSA showed no difference between CON and ECC training. Both training modalities increased the CSA in the tendon’s proximal region, which corresponded to the biopsied site; this region of the patellar tendon has previously been reported to increase in size in response to mechanical loading [37, 38]. This tendon size increase would at least partly be accounted for by hypertrophy of the tendon, as suggested via the increased expression of COL1A1 and the increase in tendon FSR that occurred in both groups. However, it should also be considered that the patellar tendon’s proximal region in particular is subjected to large compressive loading due to it wrapping around the patellar bone [39, 40]. Compressive loads are linked with the expression of large aggregating proteoglycans such as versican and biglycan, which increase water content in the tendon to provide greater resistance to compression.

### Tendon tissue remodelling following 4- and 8-week eccentric or concentric training in older males

While RE has been shown to improve tendon mechanical properties [25] and induce structural changes in humans [2], the specific adaptations with exercise in older individuals, and especially the mechanisms responsible for these changes, remain incompletely understood. In the present study, there were relatively few altered genes following either 4 or 8 weeks ECC or CON training in older males, compared to the young group. However, despite transcriptional changes indicating reduced responsiveness of training in older adults, protein synthesis responses with training were similar for both age groups. As with the ECC versus CON comparisons, there was no relation between gene expression responses to training in the older group, and protein synthesis responses, although temporal differences in gene responses could account for observed differences. Overall, the present work demonstrates that similar adaptive changes, in terms of tendon protein synthesis, were observed in healthy older individuals to younger adults. Similarly, we also observed no change in proximal tendon CSA. This is in contrast to the younger groups, but there are some potential explanations as to why this is the case. It is possible that as the elderly individuals have a higher proximal tendon CSA value at baseline; while an increase in proximal CSA is possible in the young, this may not the case in the elderly groups. Indeed, larger tendon CSA values have previously been reported in elderly compared to younger individuals [41, 42], likely due to prolonged habitual loading.

This study advances our understanding on the mechanisms of tendon collagen remodelling with exercise and age; however, our study is not without some limitations. Due to the complexity and extent of collagen crosslinking, there is evidence suggesting the existence of both dynamic and inactive collagen pools within ECM, resulting in incomplete turnover [43, 44]. This has been demonstrated in the liver and muscle of mice [43, 44], yet complete turnover has been predicted in rodent bone collagen measurements [45]. However, due to the very slow turnover rate of collagen in ECM tissues, these primarily rely on predicted plateaus, and therefore extremely long labelling studies are required to fully determine the size of any inactive collagen pool, especially in humans. That being said, the possible impact of inactive and heterogeneous collagen pools on turnover measurements needs to be acknowledged.

Our data in human patellar tendon represents initial tracer incorporation (~ 5% fraction new) and only follows the very early phase of a rise to plateau curve; we believe in this early phase that it is both valid and appropriate to calculate FSR assuming 100% renewal. Currently, there is no human data that defines the size or relative proportion of inactive and dynamic collagen pools, nor how they might change in response to a chronic intervention. Though we would postulate that any increase in the proportion of the active pool, e.g. in response to loading/exercise, would result in greater overall incorporation of tracer resulting in a greater FSR, a reduction in the active pool would result in less tracer incorporation and an apparent reduction in FSR. Given our intervention is relatively short in relation to the time involved for the tendon collagen pool to fully turnover, we contend that the data reflect the physiological responses of tendon to eccentric and concentric exercise. There is evidence from human Achilles tendon studies that some tendon made during early growth remains throughout life [46]. However, tendon has demonstrated active collagen synthesis at rest, with an increase in the rate of synthesis in response to exercise [29]. That being said, if large proportions of tendon are inactive, then FSR would be increased [43], yet comparisons across conditions would still be warranted.

## Conclusions

In summary, we report a general downregulation of tendon collagen/cross-linking genes, and specific proteoglycan genes in older age, while there was evidence of increased tendon proline hydroxylation with age. While it appeared that the older tendon was less responsive to exercise training, in terms of a molecular remodelling perspective, protein synthesis was similar for both age groups. Finally, changes in remodelling with exercise appeared to occur early in tendon (with few changes seen with 8 weeks of training), and although ECC appeared to induce greater remodelling of tendon than CON at the molecular level, there was no observable difference in terms of protein turnover between training types. Nevertheless, similar responses of protein turnover with ECC training relative to CON, in relation to the lower metabolic cost and greater application of strain that can be achieved with this training type, could provide support for an overall more beneficial response of ECC training for tendon tissue. The present investigation provides novel insights into the influences of longer-term training on cellular responses in the human tendon. Future work should aim to investigate whether/how these cellular changes to tendon may/may not translate to functional outcomes with training in older individuals.

## Supplementary information

Below is the link to the electronic supplementary material.

Supplementary file1 (DOCX 235 KB)

Supplementary file2 (DOCX 444 KB)

Supplementary file3 (DOCX 475 KB)
