# Supplementary material for: Metabolic and molecular responses of human patellar tendon to concentric- and eccentric-type exercise in youth and older age
Source: GeroScience. 2022 Aug 11;45(1):331–44. doi: 10.1007/s11357-022-00636-x (PMC9886711; doi:10.1007/s11357-022-00636-x)
Supplement: Supplementary file 1 — Supplementary file1 (DOCX 235 KB) [file 11357_2022_636_MOESM1_ESM.docx]

**
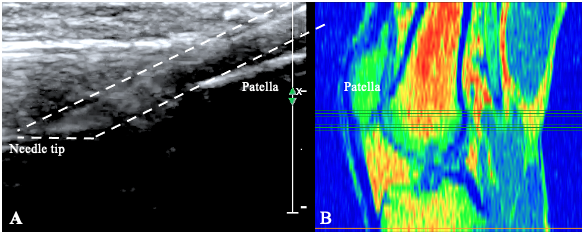
**

**Supporting Figure 1: Representative image of the patellar tendon biopsy location captured via ultrasound.** (A) demonstrates the location at which the tendon biopsy was obtained with the dashed line highlighting the biopsy needle. (B) demonstrates the matched location upon the MRI scan whereby proximal tendon CSA was calculated. The top green line represents the first measurement, whilst the second green line represents the final measurement.

**Article name**: Metabolic and molecular responses of human patellar tendon to concentric and eccentric-type exercise in youth and older age

**Journal name**: GeroScience

**Author names**: Hannah Crossland, Matthew S Brook, Jonathan I Quinlan, Martino V Franchi, Bethan E Phillips, Daniel J Wilkinson, Constantinos N Maganaris, Paul L Greenhaff, Nathaniel J Szewczyk, Kenneth Smith, Marco V Narici, Philip J Atherton

**Corresponding author affiliation and email address**: The University of Nottingham, Philip.Atherton@nottingham.ac.uk
