# Supplementary material for: Metabolic and molecular responses of human patellar tendon to concentric- and eccentric-type exercise in youth and older age
Source: GeroScience. 2022 Aug 11;45(1):331–44. doi: 10.1007/s11357-022-00636-x (PMC9886711; doi:10.1007/s11357-022-00636-x)
Supplement: Supplementary file 2 — Supplementary file2 (DOCX 444 KB) [file 11357_2022_636_MOESM2_ESM.docx]

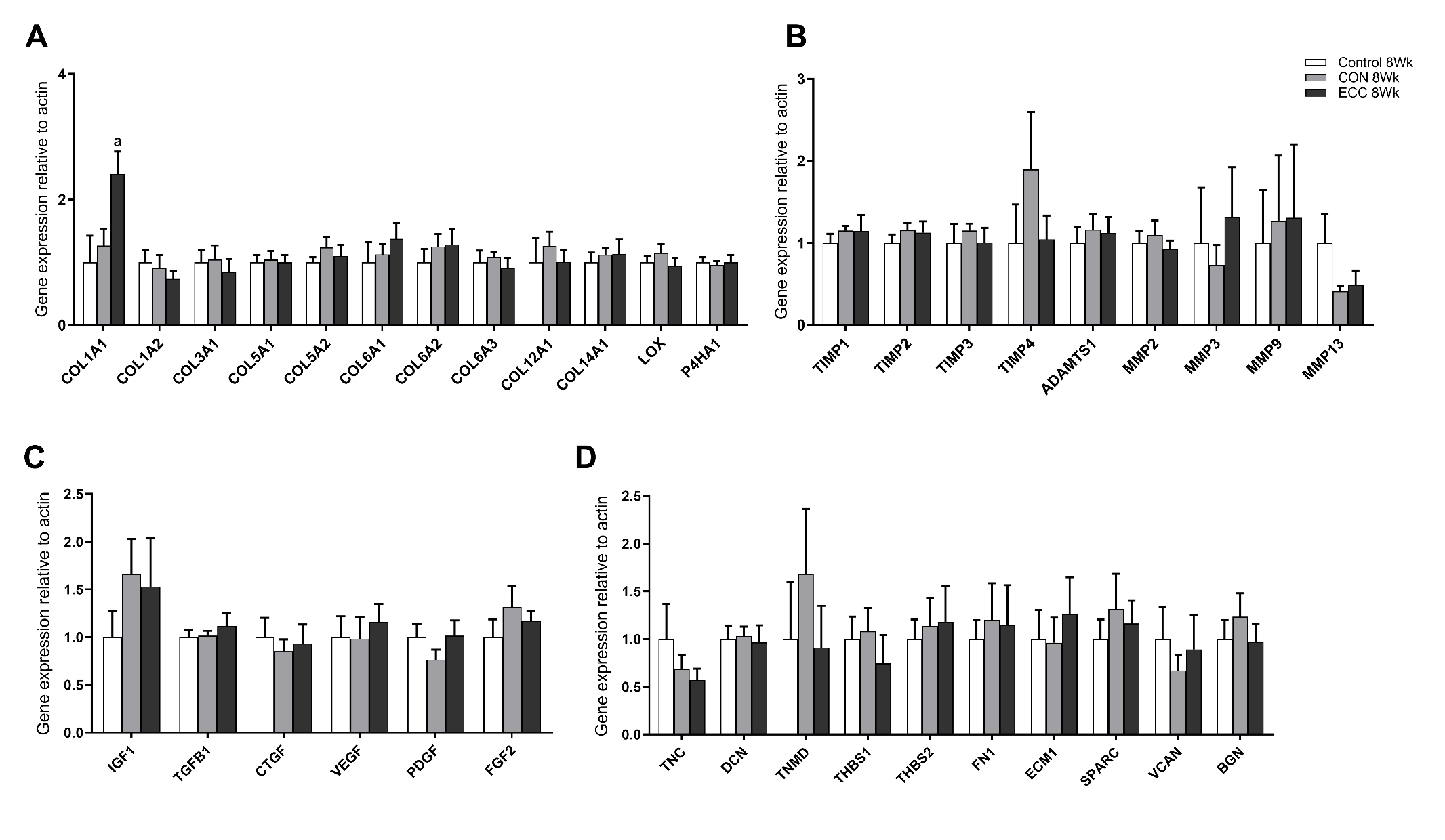


**Supporting Figure 2. Expression of extracellular matrix (ECM)-related and regulatory growth factor genes in young individuals following 8 weeks eccentric (ECC) or concentric (CON) training in tendon tissue.** Expression of genes related to collagen proteins (A), ECM remodelling (B), regulatory growth factors (C) and structural ECM-related proteins and proteoglycans (D) were compared in young individuals following 8 weeks ECC or CON training in tendon tissue, with data normalized to actin expression and presented as fold change versus controls (n=9 per group). Results are displayed as mean+SEM. ^a^*P*<0.05 versus control group.

**Article name**: Metabolic and molecular responses of human patellar tendon to concentric and eccentric-type exercise in youth and older age

**Journal name**: GeroScience

**Author names**: Hannah Crossland, Matthew S Brook, Jonathan I Quinlan, Martino V Franchi, Bethan E Phillips, Daniel J Wilkinson, Constantinos N Maganaris, Paul L Greenhaff, Nathaniel J Szewczyk, Kenneth Smith, Marco V Narici, Philip J Atherton

**Corresponding author affiliation and email address**: The University of Nottingham, Philip.Atherton@nottingham.ac.uk
